# The Treatment of Insomnia

**Published:** 1881-02

**Authors:** 


### THE TREATMENT OF INSOMNIA.

The following suggestions are made by Dr. James More, in the *Practitioner*, Dec., 1880: —

During sleep the cutaneous capillaries are in a state of dilatation, so as to ensure that degree of cerebral anæmia which the physiologist tells us is a necessity to the presence of the "drowsy god." This flushing of the skin surface with blood, with its consequent cerebral anæmia, is fostered and provided for in the warm bed-clothes which the habit of ancestral civilized society has made almost an instinct, and stamped as a physiological necessity.

The physician, when called upon to prescribe for insomnia, meets with it in various aspects, but in a large number of instances it is due to direct or indirect hyperæmia of the brain.

He sees it in the disturbed circulation of the bilious or gouty old gentleman; in the anæmic girl or the plethoric matron; in the patient with cold feet, or in the one again with flushed face and throbbing temples. In each and all the fundamental condition of sleep — cerebral anæmia — is absent, and what is wanting is something that will unload the cerebral vessels of their surplus blood. The surplus may be due either to arterial hyperæmia, or to venous engorgement, and the skillful physician will follow the indication.

Dilate the cutaneous capillaries, and we withdraw the surplus blood from the surcharged brain to the skin. A glass of hot grog at bedtime, a foot-bath of hot water, with or without mustard, bottle of hot water to the feet, may be all that is necessary to dilate and flush the peripheral arterioles. Such, in many cases, act as true, safe and scientific hypnotics.

Failing in this direction, we take to those remedies which the therapeutist tells us dilate the cutaneous capillaries; and foremost and safest, and most reliable among these we name bromide of potassium. This, with a little tension help from digitalis, forms an almost certain sleeping draught in most of the above conditions. A draught as follows might be given: —

| | | |
|---|---|---|
| R. | Bromid. potass., | gr. xxv |
| | Tinct. digitalis, | gtt. xv. |

This failing, the addition of even so small a dose as one drachm of syrup of chloral hydrate will "make all the difference between success and failure." Of course, in many cases a slight anodyne effect is wanted to complete any special draught, and the addition of a few drops of chlorodyne or Battley's sedative, besides doing this, seems to accentuate or intensify the action of the vaso-dilators.

This was forcibly illustrated in the case of a patient I saw not long ago, where almost absolute sleeplessness had existed for some weeks, the result of mental excitement and worry. He had been taking as much as six drachms of syrup of chloral nightly, without effect, and viewing it clearly as a case of cerebral hyperæmia, I prescribed the following combination, which acted at once and with marked and decided effect: —

| | | |
|---|---|---|
| R. | Bromid. potass., | gr. xx |
| | Tinct. digitalis, | gtt. xv |
| | Syrup. chloral hyd., | oz. j |
| | Liq. opii sed., | gtt. v. |

We do not mean to aver that such a combination acted solely on the hydraulic principle, or had no decided neurosal effect on the brain cells, but what we hold is that its power in dilating the cutaneous arterioles, and thus providing for cerebral anæmia, is its chief *modus operandi.*

